# Supplementary material for: A spike-timing-dependent plasticity rule for dendritic spines
Source: Nat Commun. 2020 Aug 26;11:4276. doi: 10.1038/s41467-020-17861-7 (PMC7449969; doi:10.1038/s41467-020-17861-7)
Supplement: Supplementary file 1 — Supplementary Information [file 41467_2020_17861_MOESM1_ESM.pdf]

SUPPLEMENTARY INFORMATION

A spike-timing-dependent plasticity rule for dendritic spines

Authors

Sabrina Tazerart[1,2,3], Diana E. Mitchell[1,2,3], Soledad Miranda-Rottmann[1,2], and Roberto Araya[1,2,✉]

Affiliations

[1]Department of Neurosciences, Faculty of Medicine, University of Montreal, Montreal, QC, Canada

[2]The CHU Sainte-Justine Research Center, Montreal, QC, Canada

[3]These authors contributed equally to this work

✉Corresponding author: Roberto Araya Ph.D. Associate Professor, Department of Neurosciences, Faculty of Medicine, University of Montreal, CHU Ste-Justine Research Center, 3175 Côte Sainte-Catherine, Montréal, Québec, Canada H3T 1C5, T: 514-343-7815, e-mail: roberto.araya@umontreal.ca

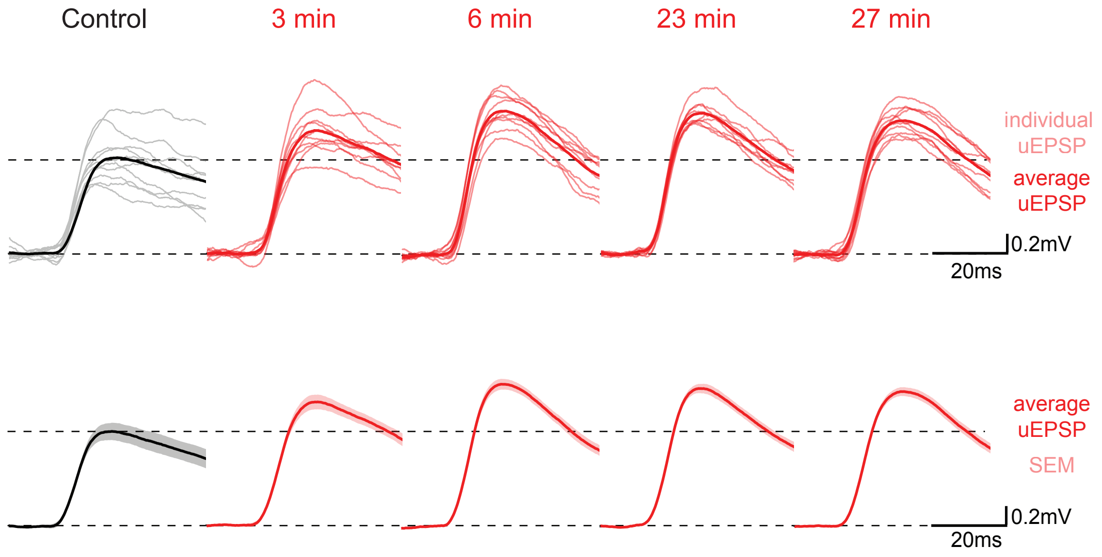

Supplementary figure 1: Individual uEPSP traces following the induction of t-LTP in single dendritic spines. Experiments were performed by 2P activation of spines from basal dendrites in L5 pyramidal neurons and uEPSP were recorded in the soma. Gray lines show individual (top) and black lines the average (top and bottom) uEPSP traces before any STDP induction protocol was applied. Light red lines show individual (top) and solid red lines average (top and bottom) uEPSP traces following a pre-post pairing protocol of +13 ms. Shaded area represents SEM (bottom).

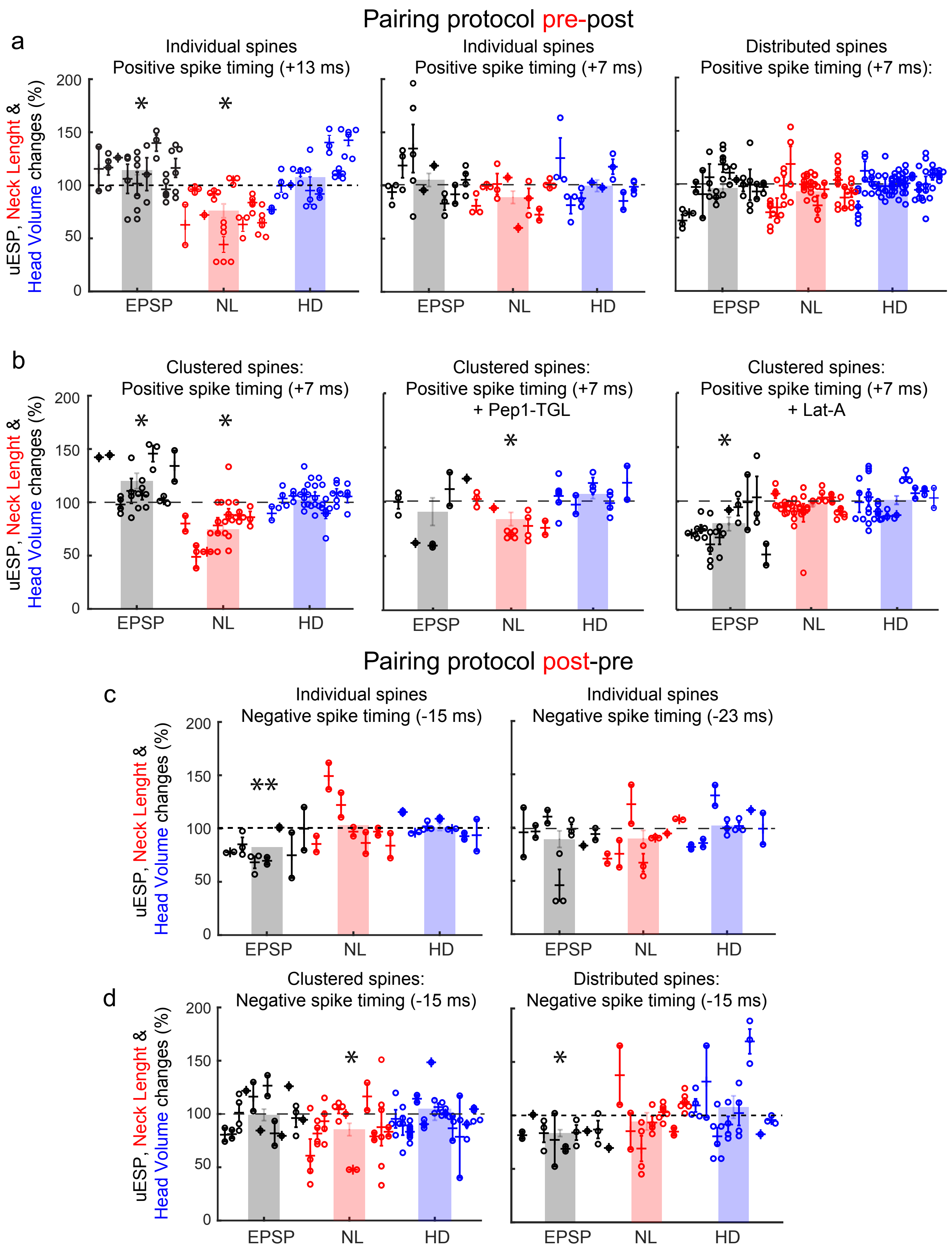

Supplementary figure 2: Average of all values obtained following STDP induction for uEPSP amplitude, neck length and head volume. Plots showing all the data points for change in uEPSP (black bars and dots), neck length (red bars and dots) and head volume (blue bars and dots) obtained following STDP induction using positive spike timings **a** in single and distributed spines, positive spike timings **b** in clustered spines, negative spike timings **c** in single spines, and negative spike timings **d** in clustered and distributed spines. Each column represents data points from a single experiment. Crosses and errors bars indicate the average and SEM for each individual experiment, while the shaded bar graphs and error bars represent the average of the mean and SEM from each individual experiment. *P < 0.05; **P < 0.01; two-sided Wilcoxon test. For exact P values see Supplementary Table 1. Note that statistical significance remains the same whether we consider average uEPSP and morphology changes 15-25 minutes (Figure 1-5) or the average of all values obtained following STDP induction for uEPSP amplitude, neck length and head volume.

Group	uEPSP *p* (n)	Neck length *p* (n)	Head volume *p* (n)
Individual spines: Positive spike timing (+13 ms)	0.012 (9)	0.012 (9)	0.496 (9)
Individual spines: Positive spike timing (+7 ms)	0.55 (8)	0.25 (8)	0.94 (8)
Distributed spines: Positive spike timing (+7 ms)	0.34 (13)	0.062 (20)	0.44 (26)
Clustered spines: Positive spike timing (+7 ms)	0.023 (8)	0.004 (10)	0.070 (16)
Clustered spines: Positive spike timing (+7 ms) + Pep1-TGL	0.625 (5)	0.015 (8)	0.69 (10)
Clustered spines: Positive spike timing (+7 ms) + Lat-A	0.012 (8)	0.068 (14)	0.95 (16)
Individual spines: Negative spike timing (-15 ms)	0.031 (7)	0.94 (7)	0.94 (7)
Individual spines: Negative spike timing (-23 ms)	0.16 (7)	0.22 (7)	0.94 (7)
Clustered spines: Negative spike timing (-15 ms)	1 (12)	0.06 (17)	0.69 (24)
Distributed spines: Negative spike timing (-15 ms)	0.016 (8)	0.36 (14)	0.80 (16)

Supplementary table 1: Statistical information associated with Supplementary Figure 2. All tests are two-sided Wilcoxon test.

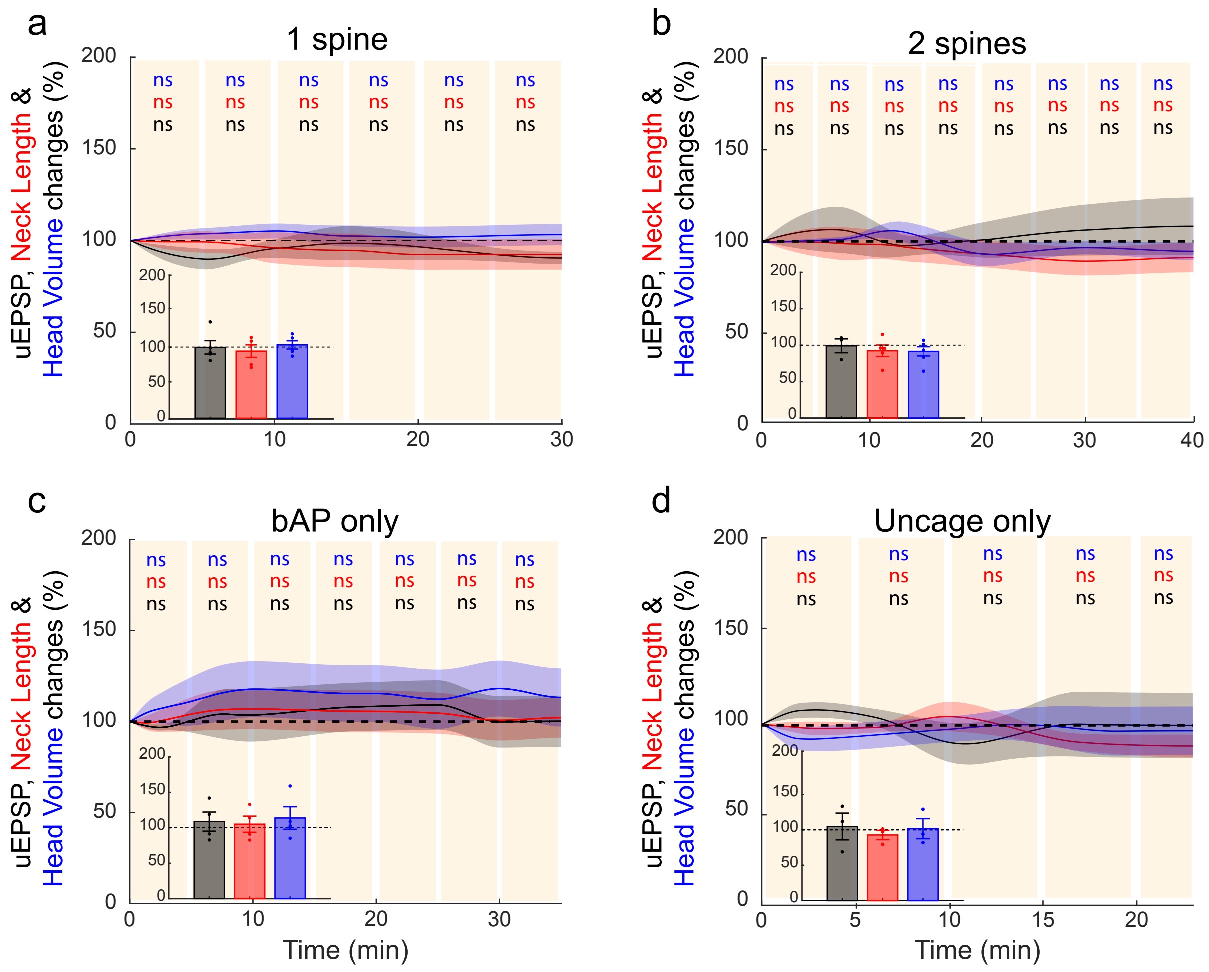

Supplementary figure 3: Control experiments showing the stability of uEPSP amplitude and spine morphology over time when no pairing protocol was applied. a-b Time course of uEPSP amplitude (black line), neck length (red line) and spine head volume (blue line) over the course of ~30 min after uncaging in **a** one (n = 5 spines) and **b** two spines (n = 3 spine pairs) approximately every 5 minutes, without inducing any STDP protocol. Insets show changes in uEPSP amplitude (black bar and dots), neck length (red bar and dots) and head volume (blue bar and dots) averaged over 15-25 min from the first stimulation without any STDP protocol. **c-d** Time course of uEPSP amplitude (black line), neck length (red line) and spine head volume (blue line) over the course of ~30 min **c** following bAP only (n = 4 spines) and **d** synaptic stimulation only (uncage; n = 3 spines). Insets show the changes in uEPSP amplitude (black bar and dots), neck length (red bar and dots) and head volume (blue bar and dots) averaged over 15-25 min following bAP or synaptic stimulation. ns, not significant, one-way repeated measures ANOVA followed by post hoc Dunnet's test. Shaded area and error bars represent SEM.

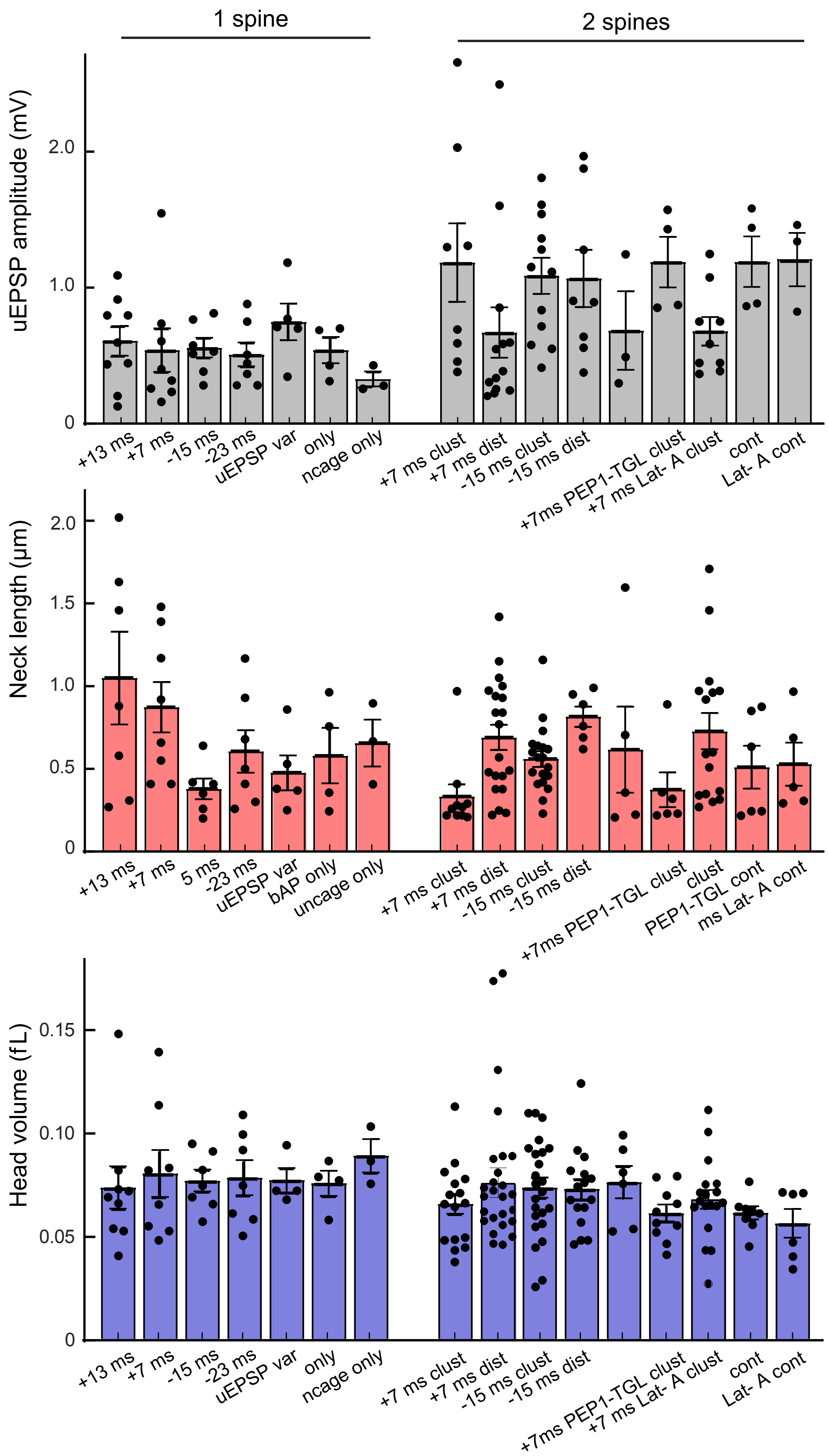

Supplementary figure 4: Absolute values for uEPSP amplitude, neck length and head volume. Bar plots showing the initial values for uEPSP amplitude (black bars), neck length (red bars) and head volume (blue bars) for each STDP protocol applied. A one-way ANOVA followed by a post hoc Tukey's multiple comparison test revealed that the uEPSP amplitude was not significantly different across all conditions when one spine ($P = 0.65$, $n = 43$ experiments) or two spines ($P = 0.22$, $n = 64$ experiments) were activated with two-photon uncaging of glutamate. A significant difference in neck length was only found between a pre-post pairing protocol of +13 ms in one spine and +7 ms in two clustered spines ($P < 0.05$, $n = 133$ spines; one-way ANOVA followed by a post hoc Tukey's multiple comparison test). The head volume across all conditions was not significantly different ($P = 0.051$, $n = 173$ spines; one-way ANOVA followed by a post hoc Tukey's multiple comparison test). uEPSP variability (var), NL var, HV var and uEPSP var 2sp, NL var 2sp, HV var 2sp correspond to the actual values for uEPSP amplitude, neck length, and head volume, respectively, from the experiment shown in Supplementary Figure 3a and b; bAP only, and uncage only correspond to the actual values for uEPSP amplitude, neck length, and head volume from the experiment shown in Supplementary Figure 3c and d, respectively. Error bars represent SEM.

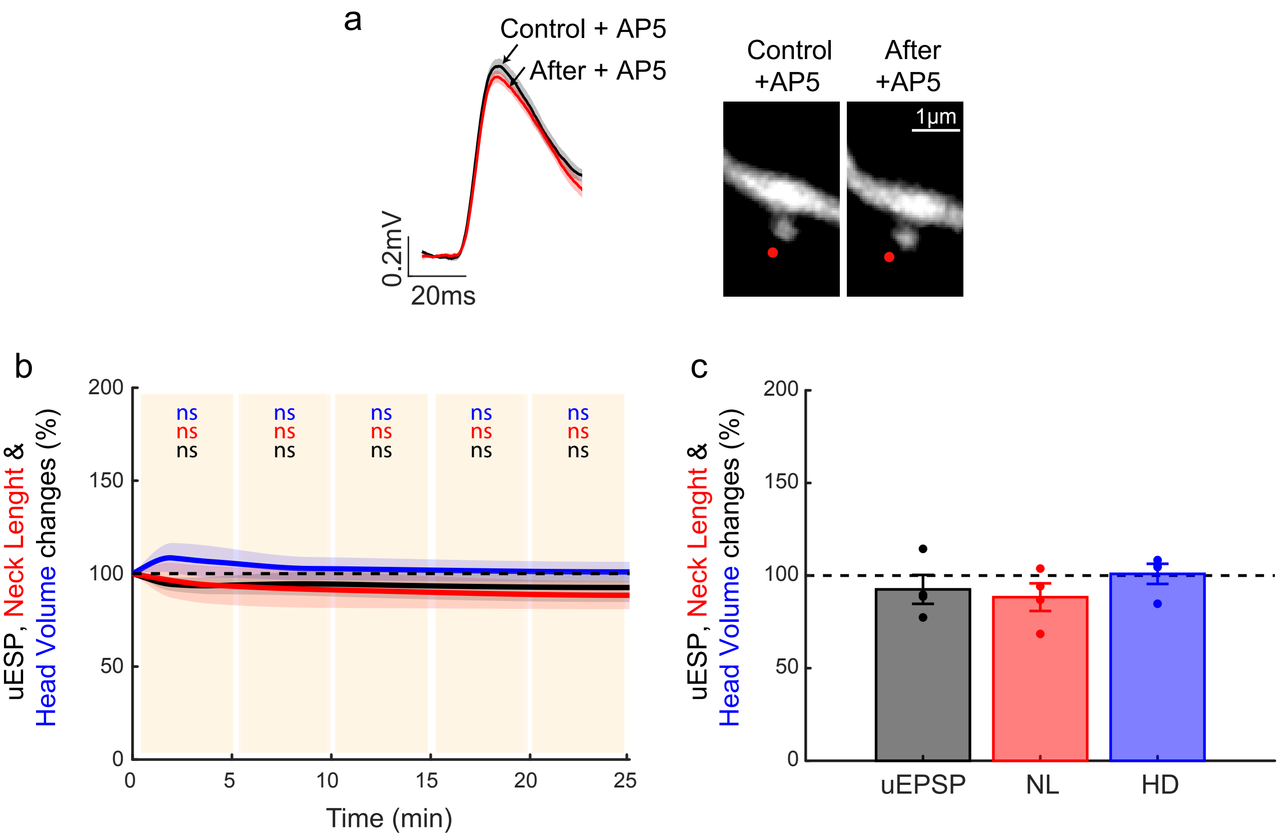

Supplementary figure 5: NMDA receptors are required for the induction of t-LTD in single dendritic spines. a Representative experiment where a single spine from a L5 pyramidal neuron basal dendrite was activated with a post-pre pairing protocol of -15 ms. Traces correspond to an average of 10 uEPSP recorded in the soma and generated by the 2P uncaging in the presence of the selective NMDA receptor antagonist AP5 (50 µM) before (control, black trace) and after the induction of t-LTP (red trace) over the indicated spine (red dot). **b** Time course of uEPSP amplitude (black line), neck length (red line) and spine head volume (blue line) changes over the course of ~30 min following STDP induction in a single spine at a post-pre timing of -15 ms in the presence of AP5 (50 µM). ns, not significant; one-way repeated measures ANOVA followed by post hoc Dunnet's test. **c** Changes in uEPSP amplitude (black bar and dots) and concomitant changes in neck length (red bar and dots) and head volume (blue bar and dots) of the activated spine 15-25 min after the induction of t-LTD at a post-pre timing of -15 ms in the presence of 50 µM AP5 (uEPSP = 92.53 ± 7.81%, P = 0.63, n = 4 spines; neck length = 88.33 ± 7.47%, P = 0.25, n = 4 spines; spine head volume = 108.08 ± 10.78%, P = 0.63, n = 4 spines, two-sided Wilcoxon test). NL = neck length, HV = head volume. Shaded area and error bars represent SEM.

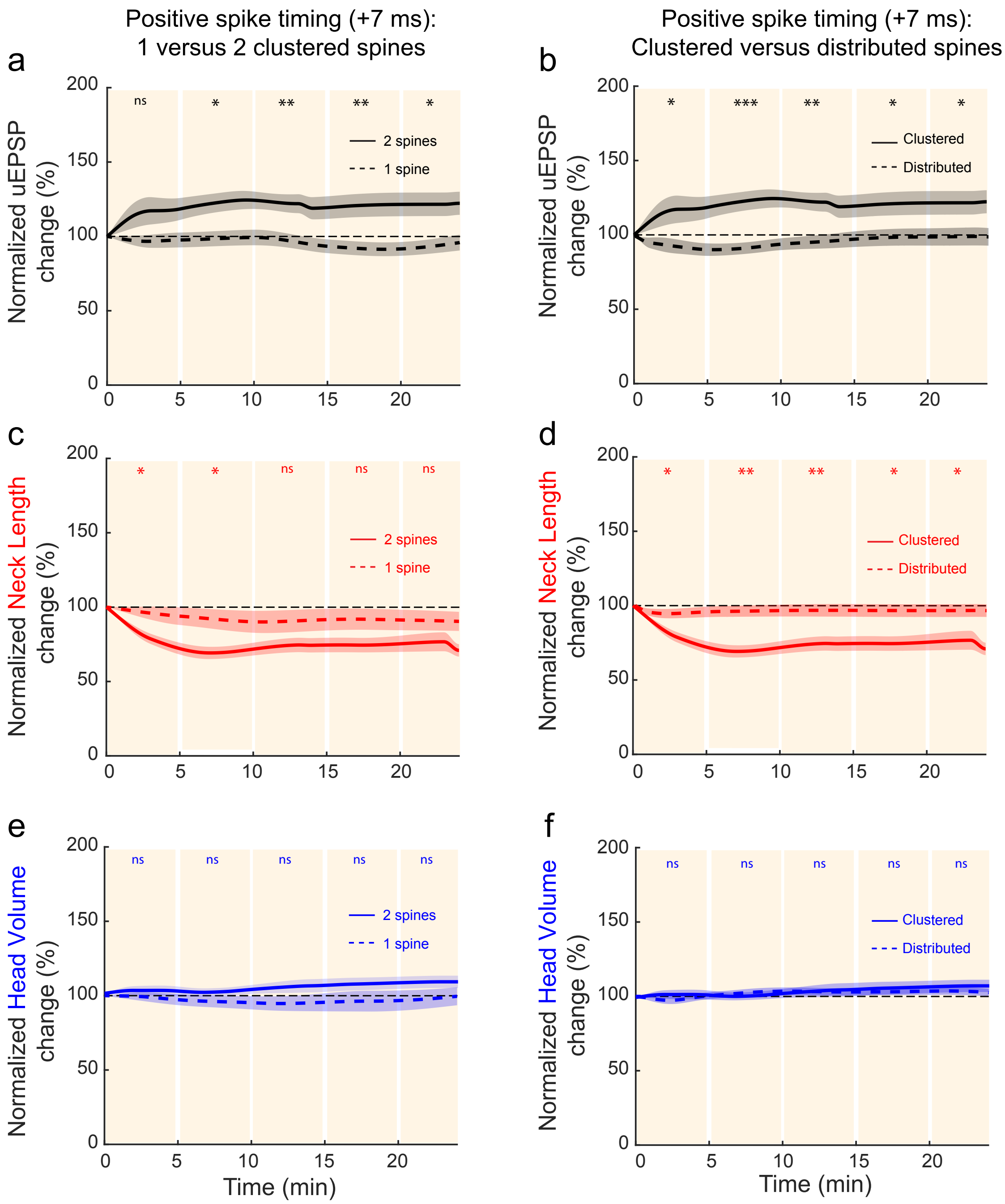

Supplementary figure 6: Induction of t-LTP in single, clustered and distributed dendritic spines at pre-post pairings of +7 ms. Comparison of the time course of **a-b** uEPSP amplitude, **c-d** neck length and **e-f** spine head volume over the course of ~25 min following STDP induction at a pre-post timing of +7 ms between individual (dashed lines in **a**, **c**, **e**) and two clustered spines (solid lines in **a**, **c**, **e**), and between two clustered spines (solid lines in **b**, **d**, **f**) and distributed spines (dashed lines in **b**, **d**, **f**). Experiments were performed by 2P activation of spines from basal dendrites in L5 pyramidal neurons and uEPSP were recorded in the soma. P = 0.01, 0.005, 0.005, 0.015 in **a** for 5-10, 10-15, 15-20, 20-25 min bins, respectively. P = 0.046, 0.002, 0.007, 0.046, 0.033 in **b** for 0-5, 5-10, 10-15, 15-20, 20-25 min bins, respectively. P = 0.012, 0.041 in **c** for 0-5, 5-10 min bins, respectively. P = 0.026, 0.00055, 0.0088, 0.015, 0.021 in **d** for 0-5, 5-10, 10-15, 15-20, 20-25 min bins, respectively. ns, not significant; *P < 0.05; **P < 0.01; ***P < 0.001; two-sided Mann Whitney test. Shaded area represents SEM.

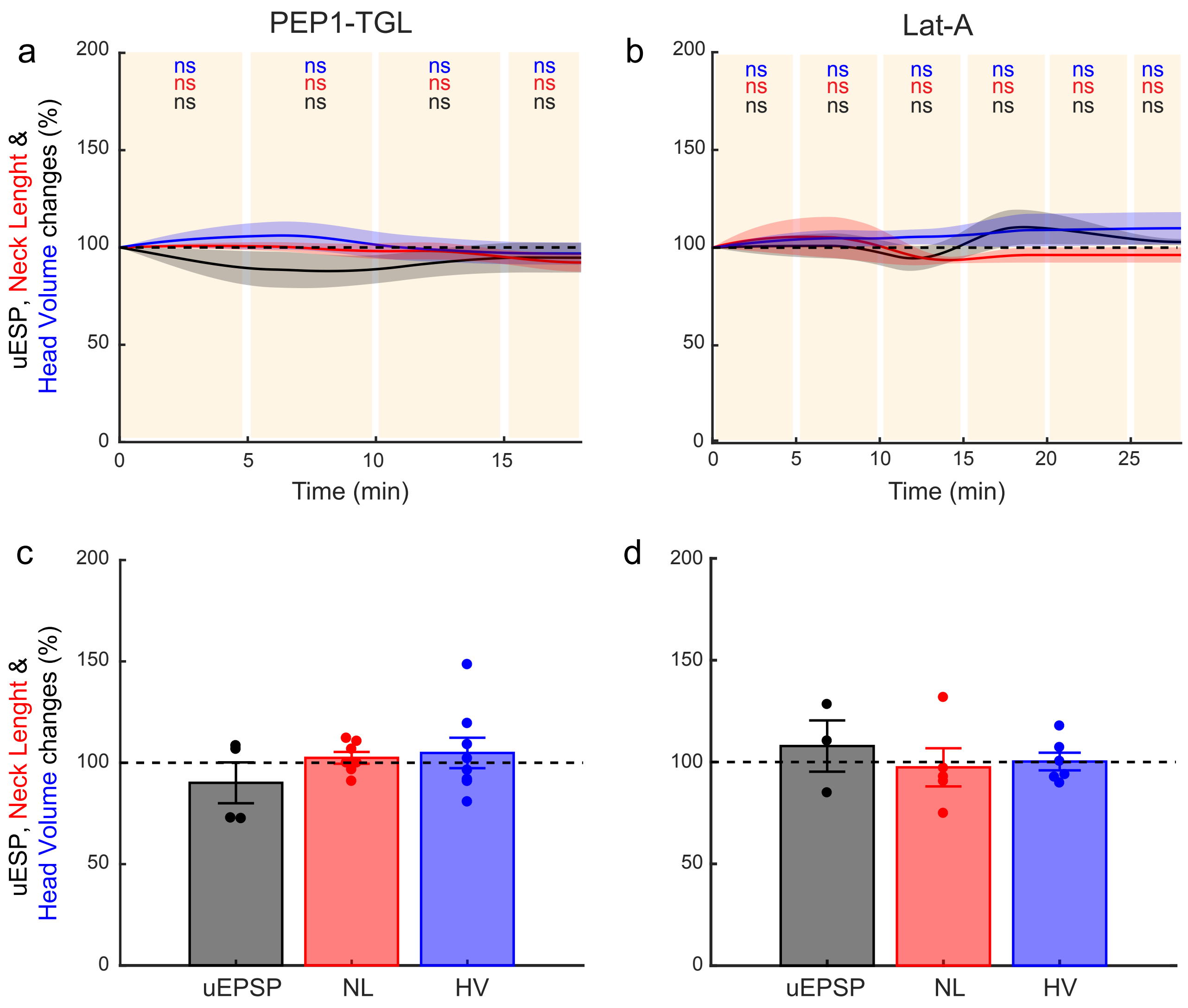

Supplementary figure 7: Effect of PEP1-TGL and Lat-A on uEPSP amplitude and spine morphology. **a-b** Time course of uEPSP amplitude (black line), neck length (red line) and spine head volume (blue line) recorded in the presence of **a** 200 µM PEP1-TGL (n = 4 spine pairs) or **b** 100 nM Lat-A (n = 3 spine pairs) without any STDP induction protocol. ns, not significant, one-way repeated measures ANOVA followed by post hoc Dunnet's test. **c-d** Changes in uEPSP amplitude (black bar and dots), neck length (red bar and dots) and head volume (blue bar and dots) of the activated spine 15-25 min following the first uncaging stimulation in the presence of **c** 200 µM PEP1-TGL (n = 4 spine pairs) or **d** 100 nM Lat-A (n = 3 spine pairs) without any STDP induction protocol. Note: Uncaging was performed in spines approximately every 5 minutes. Shaded area and error bars represent SEM.

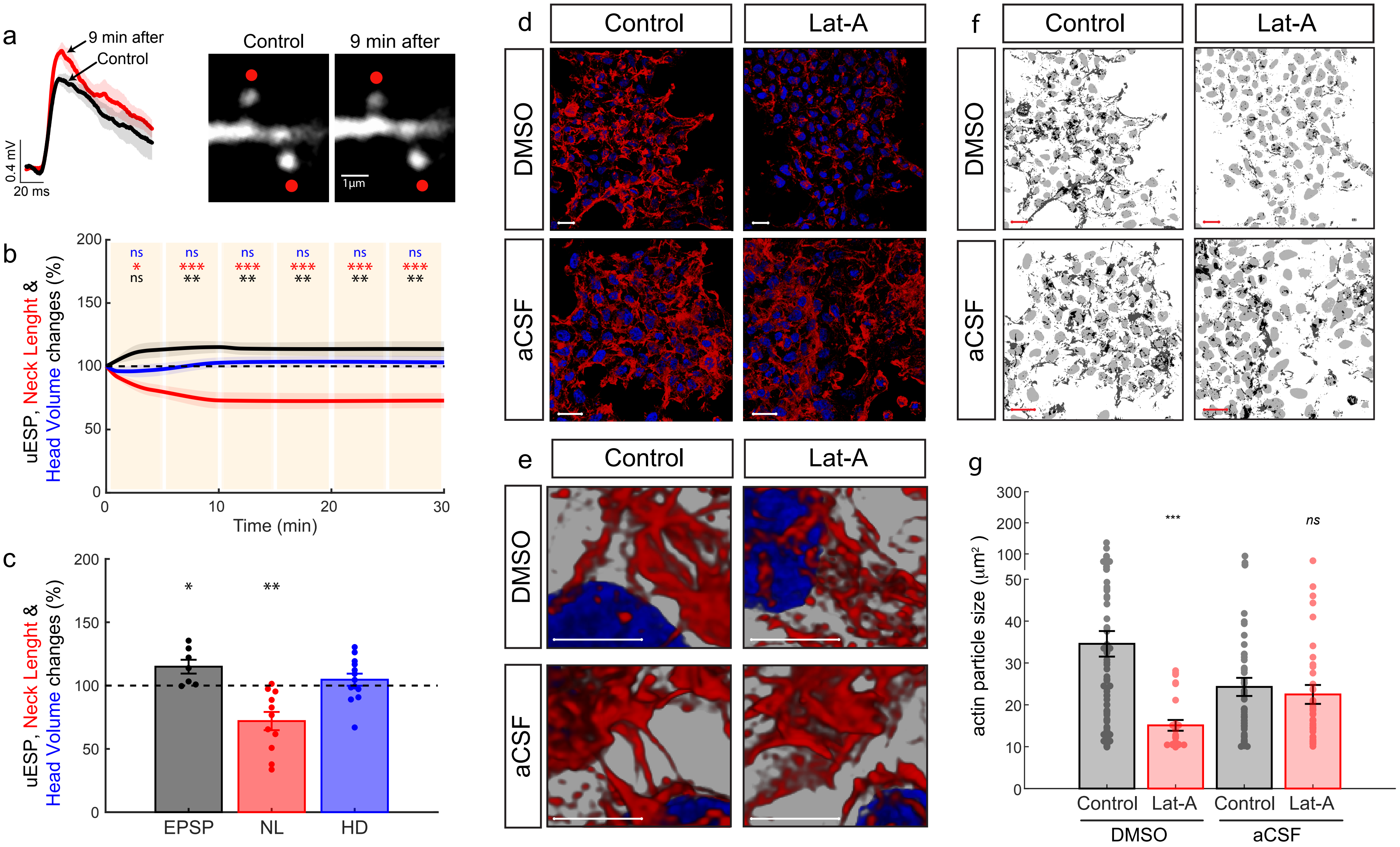

Supplementary figure 8: Effects of DMSO- or ACSF-dissolved Latrunculin A (Lat-A) on t-LTP induction in L5 pyramidal neurons and actin filaments structure in 293T cells: **a** Representative experiment where neighbouring dendritic spines from a L5 pyramidal neuron basal dendrite were activated with a pre-post t-LTP protocol. Traces correspond to an average of 10 uEPSP recorded in the soma and generated by the 2P uncaging in the presence of bath-applied Lat-A dissolved in ACSF before (control, black trace) and after the induction of t-LTP (red trace) over the indicated spine (red dots). **b** Time course of uEPSP amplitude (black line), neck length (red line) and spine head volume (blue line) changes over the course of ~30 min following STDP induction in clustered spines at a pre-post timing of +7 ms in the presence of Lat-A dissolved in ACSF. uEPSP: $P = 0.003$, $n = 7$ spine pairs; NL: $P < 0.001$, $n = 11$ spines; HV: $P = 0.12$, $n = 13$ spines; one-way repeated measures ANOVA followed by post hoc Dunnet's test; ns, not significant, *$P < 0.05$, **$P < 0.01$, ***$P < 0.001$. **c** Changes in uEPSP amplitude (black bar and dots) and concomitant changes in neck length (red bar and dots) and head volume (blue bar and dots) of the activated clustered spines after the induction of t-LTP at a pre-post timing of +7 ms in the presence of Lat-A (uEPSP = 114.93 ± 5.46%, $P = 0.03$, $n = 7$ spine pairs; neck length = 65.98 ± 9.29%, $P = 0.002$, $n = 11$ spines; spine head volume = 104.65 ± 4.77%, $P = 0.34$, $n = 13$ spines, two-sided Wilcoxon test). NL = neck length, HV = head volume. **d** Confocal image z-projection showing cells treated with 100 nM Lat-A dissolved in DMSO or artificial cerebrospinal fluid (ACSF) for 30 min (scale bar=20µm) or control with the solvent alone. Fluorescent phalloidin (red) binding to filamentous actin (f-actin) and Hoechst 33342-stained (blue) nuclei are shown. A10x objective was used. **e** 3D reconstructions of z-stacks imaged with a 63x objective (scale bar=5µm). **f** The images in panel **a** after processing for quantitative analysis of f-actin. **g** The size of all the particles counted in each experimental condition was used for statistics and plotted in MATLAB custom software. Particles analyzed/number of cells in **f**; DMSO control: 1723/52, DMSO Lat-A: 1613/109, aCSF control: 1067/79, aCSF Lat-A: 910/115 from one representative experiment of three. DMSO control ($n = 76$ particles) vs Lat-A ($n = 27$ particles) = 2.37± 0.28 vs 0.92±0.06, $P < 0.001$; ACSF control ($n = 59$ particles) vs Lat-A ($n = 40$ particles) = 2.42±0.28 vs 2.10±0.25, ns, two-sided Mann Whitney test. Shaded area and error bars represent SEM.

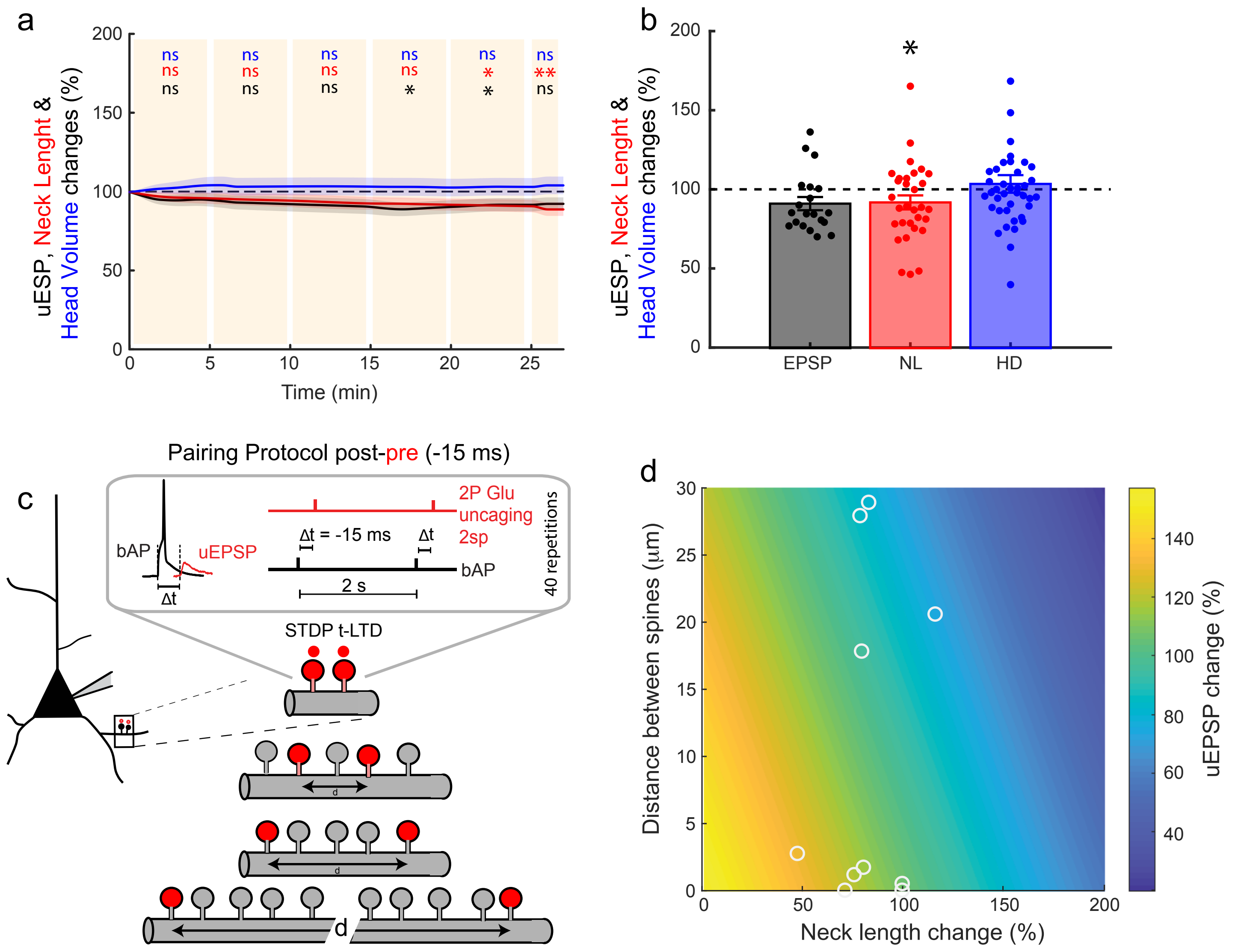

Supplementary figure 9: Induction of t-LTD and spine morphology as a function of inter-spine distance. a Time course of uEPSP amplitude (black line), neck length (red line) and spine head diameter (blue line) of the activated spines for all the inter-spine distances after the induction of t-LTD at pairings of -15 ms. uEPSP: $P = 0.04$, $n = 20$ spine pairs; NL: $P = 0.018$, $n = 30$ spines; HV: $P = 0.92$, $n = 40$ spines; one-way repeated measures ANOVA followed by post hoc Dunnet's test; ns, not significant, $*P < 0.05$. **b** Changes in uEPSP amplitude (black bar and dots), neck length (red bar and dots) and head diameter (blue bar and dots) of the two activated spines from each experiment 15-25 min after the induction of t-LTD at a post-pre timing of -15ms (uEPSP: $P = 0.067$, $n = 20$ spine pairs; neck length: $P = 0.05$, $n = 30$ spines; spine head volume: $P = 0.80$, $n = 40$ spines, two-sided Wilcoxon signed-rank test). **c** Diagram of the experimental post-pre induction protocol at pairings of -15 ms in two dendritic spines separated by different distances. **d** Color plot showing the relationship between uEPSP change (color coded) and neck length change and distance between two clustered spines following a post-pre t-LTD induction protocol. Note that when a pairing protocol of -15 ms is performed in two adjacent spines that display neck shrinkage, the result is potentiation (increase in uEPSP amplitude, more than 100%). On the other hand, when the induction protocol is performed in two spines that are further apart, without neck length changes, the result is depression (decrease in uEPSP amplitude, less than 100%). The change in uEPSP amplitude was modeled using equation 1 (described in methods). Shaded area and error bars represent SEM.

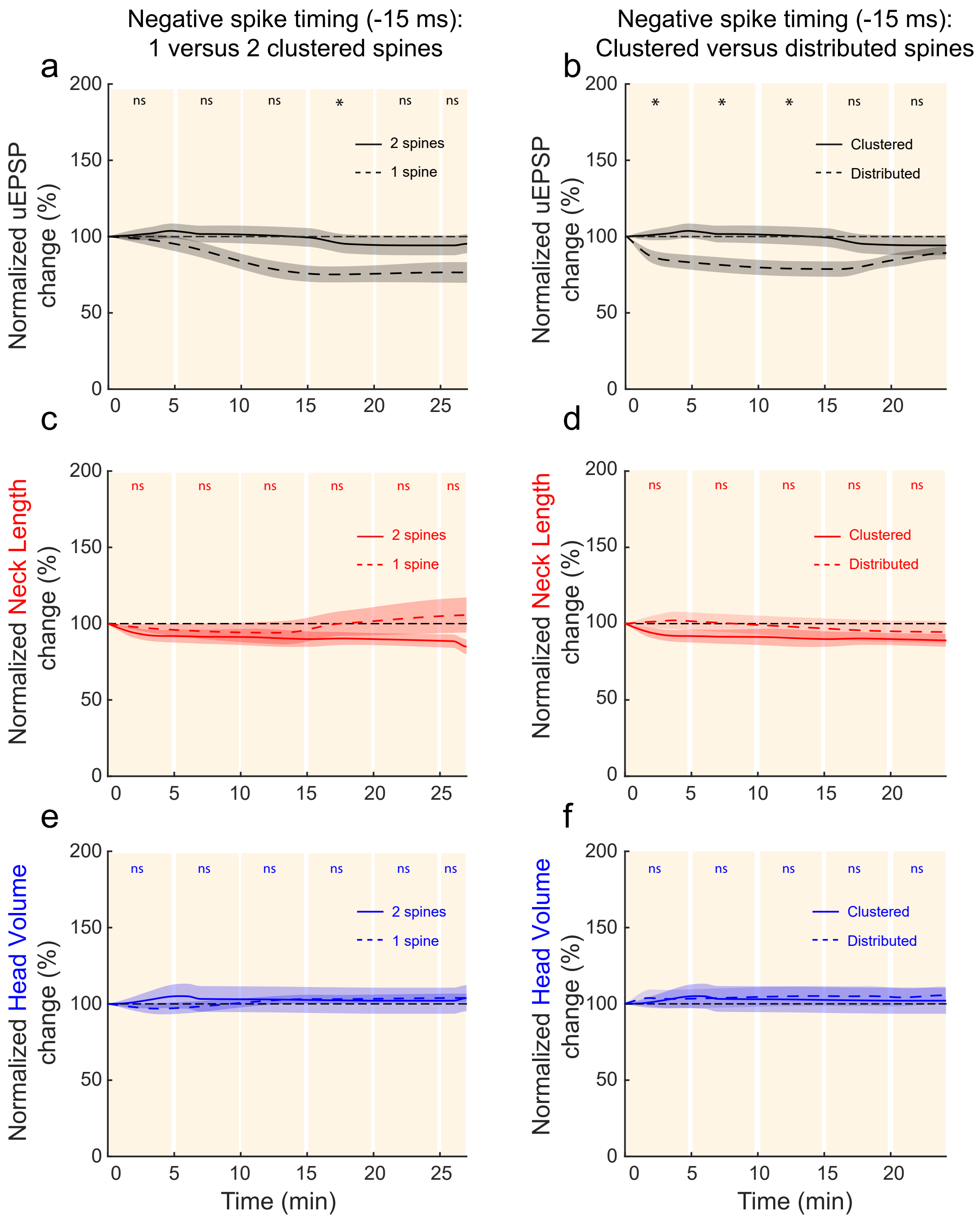

Supplementary figure 10: Induction of t-LTD in single, clustered and distributed dendritic spines. Comparison of the time course of **a**, **b** uEPSP amplitude, **c**, **d** neck length and **e**, **f** spine head volume over the course of ~25 min following STDP induction at pairings of -15 ms in individual (dashed lines in **a**, **c** and **e**) versus two clustered spines (solid lines in in **a**, **c** and **e**), and two clustered spines (solid lines in **b**, **d** and **f**) versus distributed spines (dashed lines in **b**, **d** and **f**). P = 0.021 in **a** for 15-20 min bin; P = 0.018, 0.022, 0.033 in **b** for 0-5, 5-10, 10-15 min bins, respectively. Experiments were performed by 2P activation of spines from basal dendrites in L5 pyramidal neurons and uEPSP were recorded in the soma. ns, not significant; *P < 0.05; two-sided Mann Whitney test. Shaded area represents SEM.

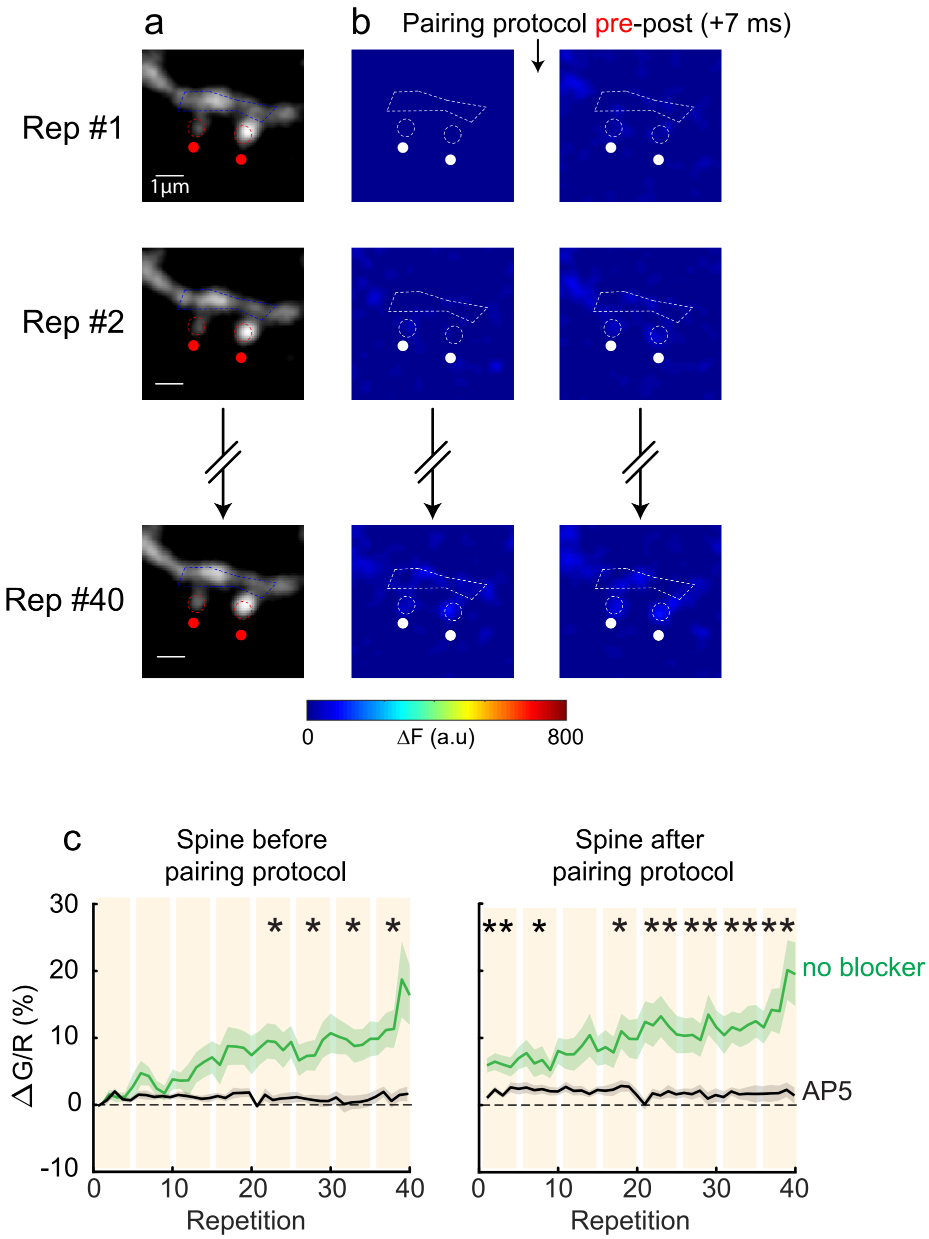

Supplementary figure 11: Induction of t-LTP in clustered spines (< 5 µm apart) generates NMDA receptor-mediated spine calcium accumulation. a Single 2P images of a spine and dendrite from a L5 pyramidal neuron loaded with Alexa 594 (100µM) and Fluo4 (300µM). Red ellipses and blue polygons indicate the ROIs selected for the calcium signal analysis. **b** Two photon calcium signal images before (left panels) and after (right panels) a pre-post pairing protocol of +7 ms. The 1st, 2nd, and 40th repetitions of the pairing protocol are shown here. The change in calcium fluorescence from baseline (ΔF) is color coded. Only positive changes in fluorescence are shown. **c** Population averages of the calcium signals (ΔG/R) measured in spines before the pairing protocol performed in 2 spines (left panels; P = 0.03, 0.02, 0.01, 0.02 for 20-25, 25-30, 30-35, 35-40 repetition bins, respectively; two-sided Wilcoxon test) and after the pairing protocol (right panels; P = 0.0017, 0.027, 0.044, 0.0062, 0.0033, 0.0018, 0.0030 for 0-5, 5-10, 15-20, 20-25, 25-30, 30-35, 35-40 repetitions bins, respectively; two-sided Wilcoxon-test) with and without AP5. ns, not significant; *P < 0.05; two-sided Wilcoxon test. Shaded area represents SEM.

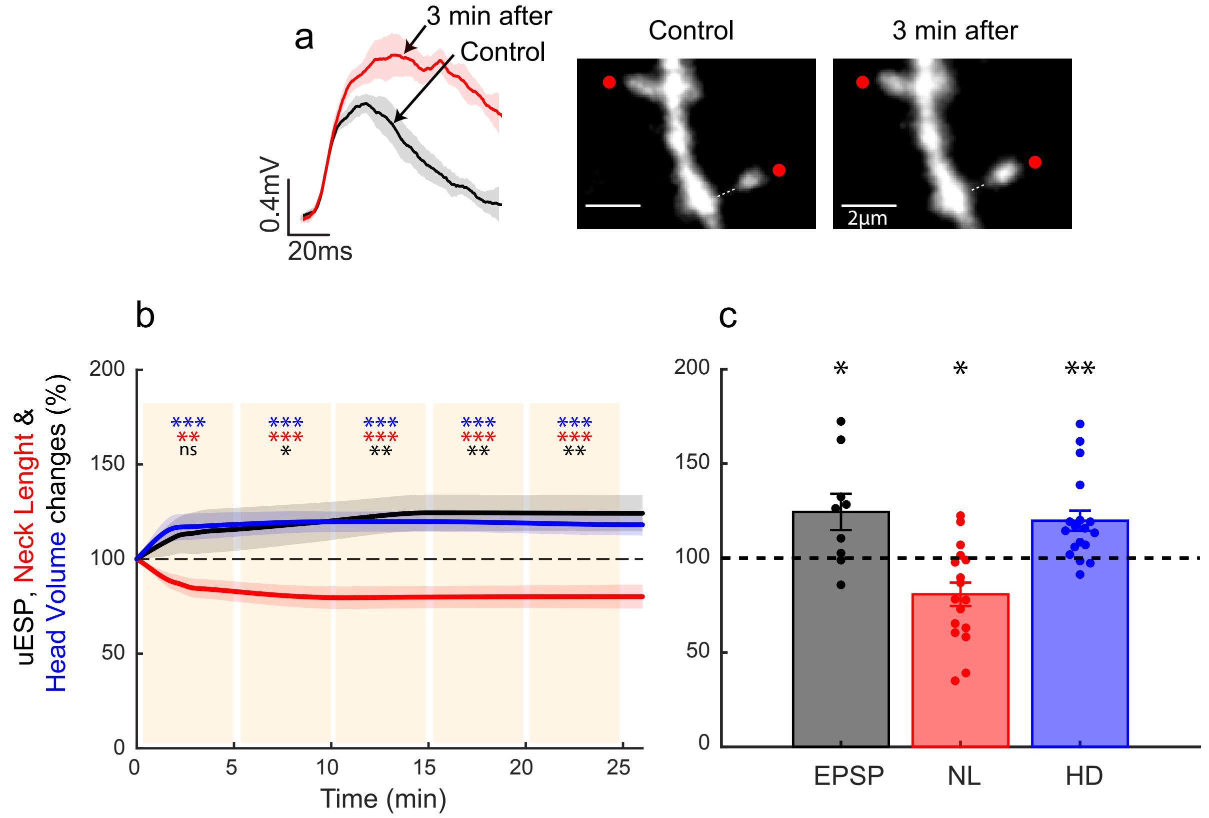

Supplementary figure 12: Induction of t-LTP in clustered spines in ACSF without Mg^{2+}. a Representative experiment where two neighbouring dendritic spines from a L5 pyramidal neuron basal dendrite were activated with a pre-post t-LTP protocol. Traces correspond to an average of 10 uEPSP recorded in the soma and generated by the 2P uncaging in ACSF containing no Mg^{2+} before (control, black trace) and after the induction of t-LTP (red trace) over the indicated spines (red dots). **b** Time course of uEPSP amplitude (black line), neck length (red line) and spine head volume (blue line) changes over the course of ~25 min following STDP induction in clustered spines at a pre-post timing of + 7 ms. uEPSP: $P = 0.0018$, $n = 9$ spine pairs; NL: $P < 0.0001$, $n = 17$ spines; HD: $P < 0.0001$, $n = 18$ spines; one-way repeated measures ANOVA followed by post hoc Dunnet's test; ns, not significant, $*P < 0.05$, $**P < 0.01$, $***P < 0.001$. **c** Changes in uEPSP amplitude (black bar and dots) and concomitant changes in neck length (red bar and dots) and head volume (blue bar and dots) of the activated clustered spines after the induction of t-LTP at a pre-post timing of +7 ms in the presence of Lat-A (uEPSP = 124.42 ± 9.62%, $P = 0.03$, $n = 9$ spine pairs; neck length = 80.70 ± 6.87%, $P = 0.007$, $n = 17$ spines; spine head volume = 120.99 ± 5.94%, $P = 0.004$, $n = 18$ spines, two-sided Wilcoxon test). NL = neck length, HV = head volume. Shaded area and error bars represent SEM.

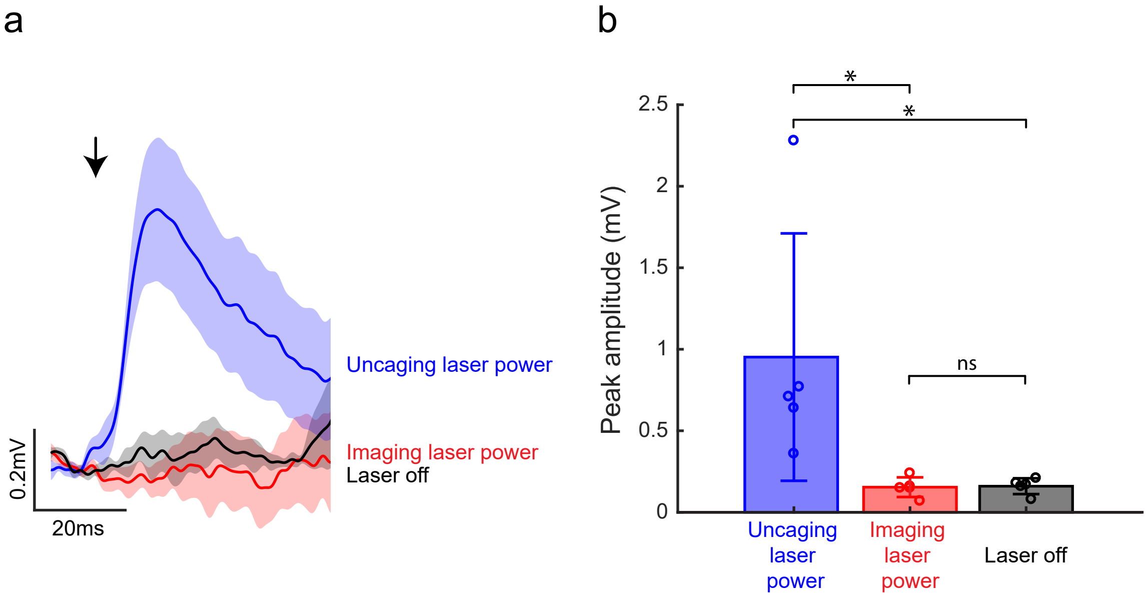

Supplementary figure 13: Imaging laser power does not cause glutamate uncaging-mediated uEPSPs in the soma of L5 pyramidal neurons. **a** Blue trace corresponds to an average of 10 depolarizations recorded at the soma triggered by uncaging glutamate next to a spine using 4 ms laser pulses of ~25-30 mW on sample at 2 second intervals (Uncaging laser power). Note the generation of a clear uEPSP. Red trace corresponds to the average voltage recorded while applying ten 4 ms laser pulses of ~5 mW on sample at 2 second intervals (Imaging laser power). Note that no uEPSPs were observed. Black trace corresponds to the average voltage recorded a second after the onset of the 4 ms laser pulses, 0 mW on sample (Laser off). Shaded area represents the SEM. **b** Plot showing peak amplitude (mV) observed after 2P uncaging of glutamate at Uncaging laser power (Blue), Imaging laser power (red), or with the 2P Laser off (black). N = 5 experiments. Uncaging versus imaging laser power: $P = 0.0079$; Uncaging laser power versus laser off: $P = 0.0079$; two-sided Wilcoxon test; ns, not significant; $^*P < 0.05$. Shaded area and error bars represent SEM.

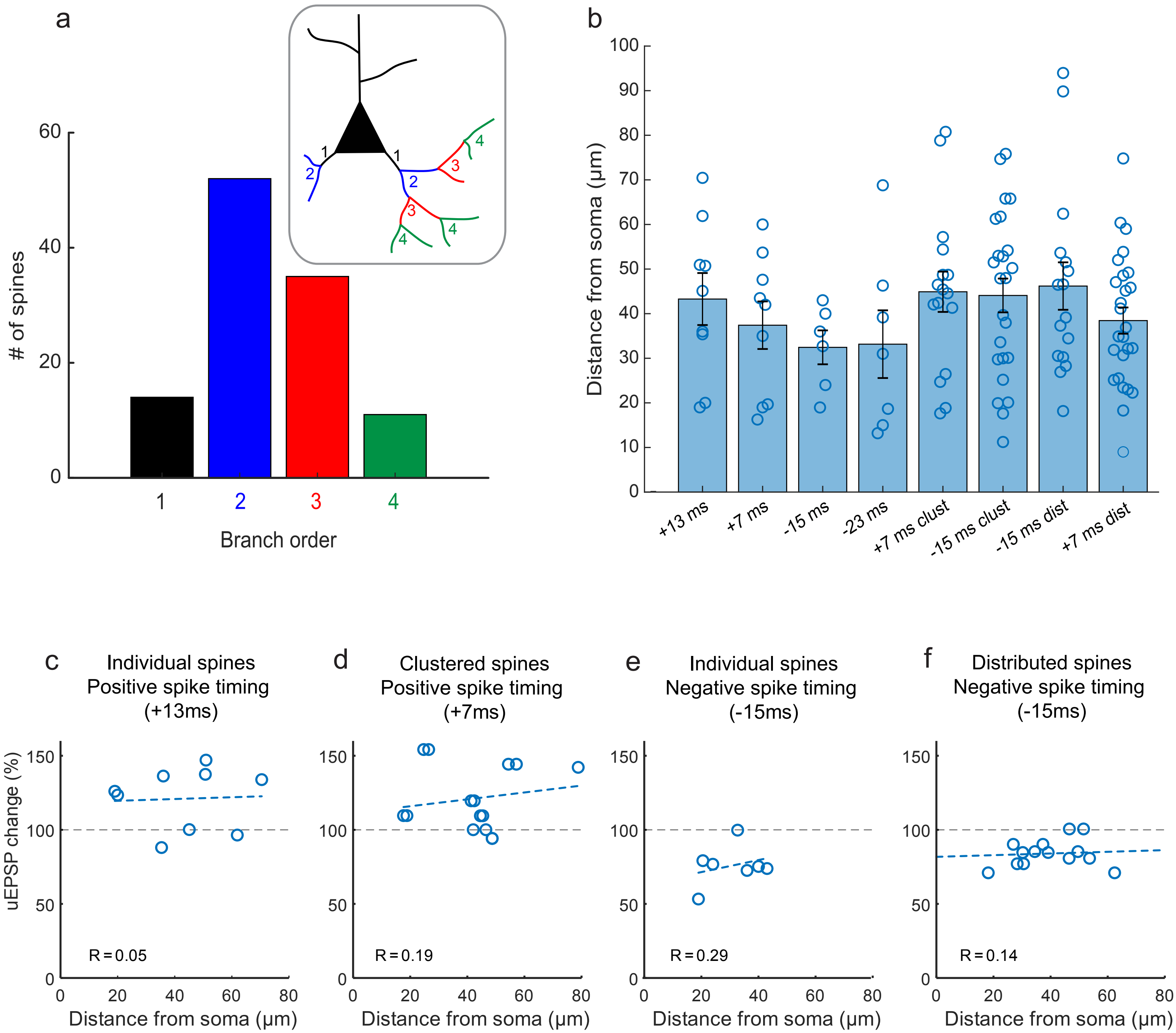

Supplementary figure 14: Morphometric analysis of spines included in this study. a Plot showing the branching order of spines that were activated in this study. A primary dendrite is one originating from the cell body (branching order labeled as "1" in inset diagram). The branching order increases with each successive branch point (when dendrite splits into two or more branches). **b** Plot showing the distance of spines from the soma for each STDP protocol that we applied. Each data point represents the distance from the soma of individual spines. No significant difference was observed across groups (40.22 ± 1.62 µm away from the soma, P = 0.55, n = 113 spines; one-way ANOVA followed by Tukey's Multiple Comparison Test). Error bars represent SEM. **c-f** Plots showing no correlation between induction of plasticity and the distance from the soma of individual spines for positive spike timing in **c** single and **d** clustered spines, and negative spike timing in **e** single and **f** distributed spines (positive spike timing: 1 spine: R = 0.05, P = 0.89, n = 9 spines; 2 clustered spines: R = 0.19, P = 0.47; n = 16 spines; negative spike timing: 1 spine: R = 0.29, P = 0.52, n = 7 spines; 2 distributed spines: R= 0.14, P = 0.60, n = 16 spines).

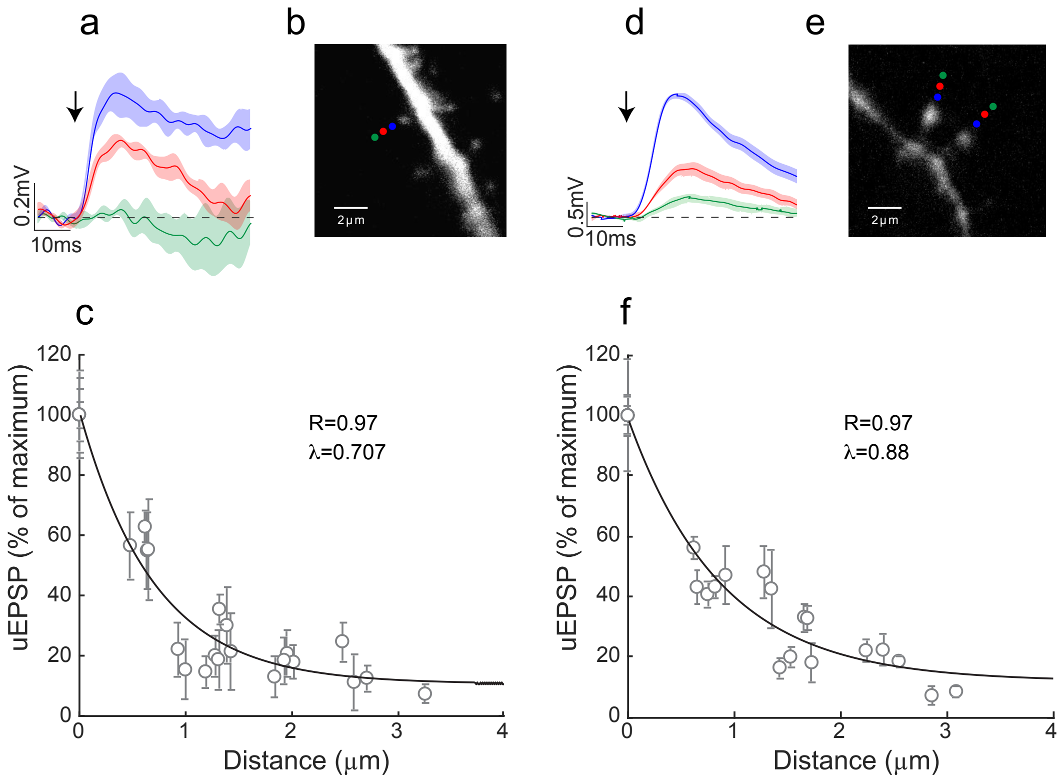

Supplementary figure 15: Spatial resolution of 2P glutamate uncaging in one and two clustered spines. Two-photon activation of single spines: **a** Example uEPSP averaged traces evoked by placing the uncaging spot at the corresponding color-coded locations shown in **b**. Each trace corresponds to an average of 10 depolarizations recorded at the soma. **c** Averaged uEPSP values (normalized to the maximum value obtained in the same experiment) as a function of distance from the closest uncaging spot in the same experiment. Two-photon activation of two clustered spines: **d** Example uEPSP averaged traces evoked by placing the uncaging spots at the corresponding color coded locations shown in **e**. Each trace corresponds to an average of ten depolarizations recorded at the soma. **f** Averaged uEPSP values (normalized to the maximum value obtained in the same experiment) as a function of distance from the closest uncaging spot in the same experiment. Shaded area and error bars represent SEM.

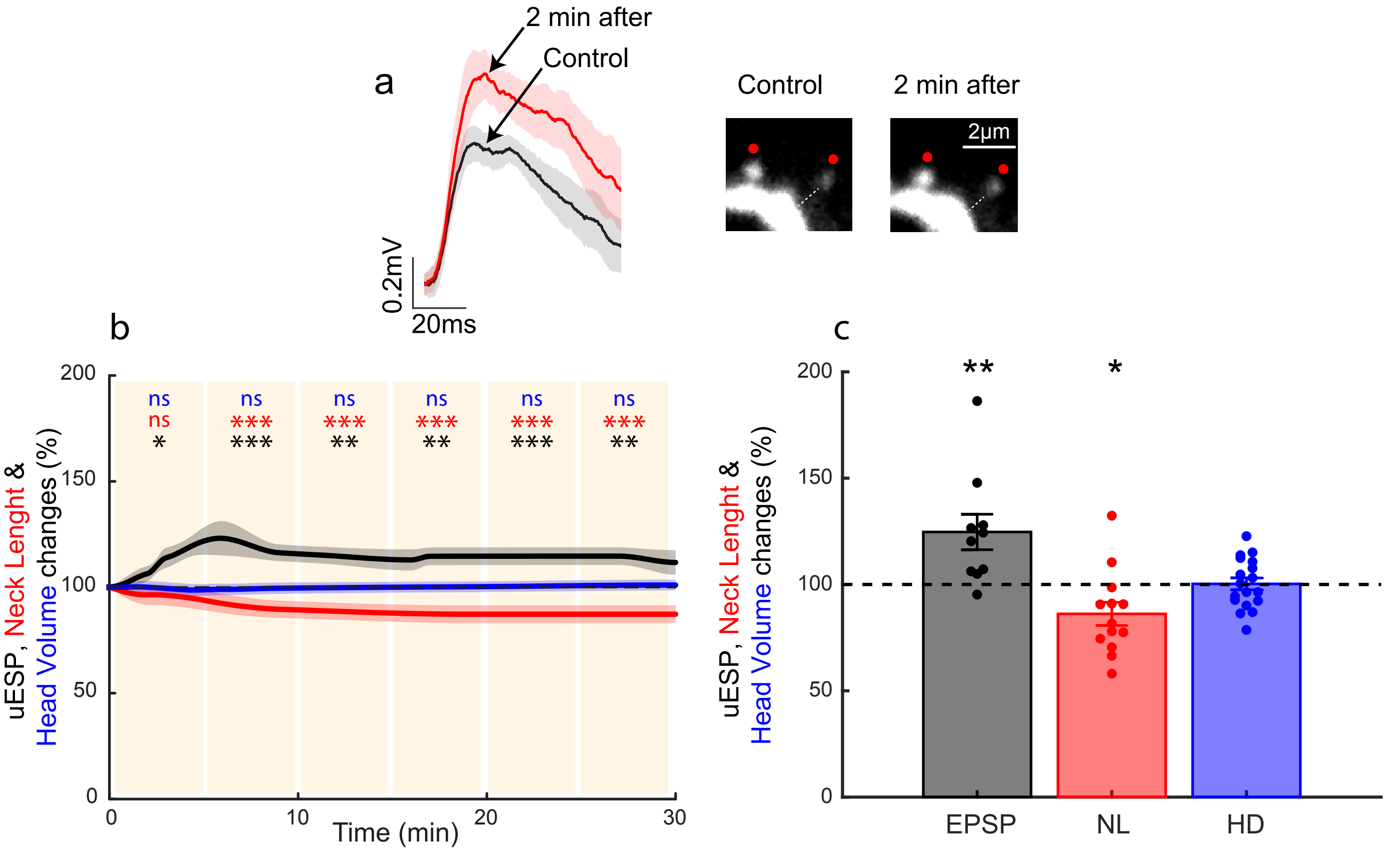

Supplementary figure 16: Induction of t-LTP in clustered spines at near-physiological temperature. **a** Representative experiment when two selected dendritic spines from a L5 pyramidal neuron basal dendrite were activated with a pre-post t-LTP protocol. Traces correspond to an average of 10 uEPSP recorded in the soma and generated by the 2P uncaging at 32ºC before (control, black trace) and two minutes after the induction of t-LTP (red trace) over the indicated spines (red dots). **b** Time course of uEPSP amplitude (black line), neck length (red line) and spine head volume (blue line) changes over the course of ~25 min following STDP induction in clustered spines at a pre-post timing of + 7 ms at 32ºC. uEPSP: $P < 0.0001$, n = 10 spine pairs; NL: $P = 0.0002$, n = 15 spines; HV: $P = 0.95$, n = 20 spines; one-way repeated measures ANOVA followed by post hoc Dunnet's test; ns, not significant; *$P < 0.05$, **$P < 0.01$, ***$P < 0.001$. **c** Changes in uEPSP amplitude (black bar and dots) and concomitant changes in neck length (red bar and dots) and head volume (blue bar and dots) of the activated clustered spines after 15-25 min of the induction of t-LTP at a pre-post timing of +7 ms (uEPSP = 113.79 ± 4.53%, $P = 0.01$, n = 10 spine pairs; neck length = 89.21 ± 5.82%, $P = 0.02$, n = 15 spines; spine head volume = 101.20 ± 2.84%, $P = 0.91$, n = 20 spines, Wilcoxon test). NL = neck length, HV = head volume.

Supplementary discussion

Induction of t-LTP-mediated spine morphological changes

We found that the induction of t-LTD was not accompanied with spine neck or head changes, which is at odds with previous findings suggesting structural changes in spine head volume during the induction of LTP or LTD [1, 2, 3]. The discrepancy between our results and those observed previously after the induction of t-LTP (head enlargement [1, 4]), LTP [2], or LTD (head shrinkage [3]) using glutamate uncaging are likely explained by methodological differences. While our data was obtained using ACSF with physiological concentrations of magnesium and calcium, those from other reports were done in low or a magnesium-free ACSF [2, 3], low calcium extracellular solution for the induction of LTD [3], or in a magnesium-free ACSF and an intracellular solution containing 5 µM actin that was required for the t-LTP-mediated spine head enlargements [1]. In fact, inducing t-LTP with a pre-post timing of +7 ms in slices that were perfused with ACSF containing no magnesium ions resulted in significantly increased uEPSP amplitudes, shrinkage of the activated spine necks, and importantly an increase in spine head size (Supplementary Figure 12).

Molecular mechanisms responsible for t-LTP in dendritic spines

What are the mechanisms responsible for the generation of t-LTP in spines? Why t-LTP induction in single and clustered spines is associated with spine neck shrinkage?

We and others have reported that LTP induction can trigger activity-dependent changes in spine neck length [5, 6] and head size [2, 6, 7], and that the amplitude of somatically recorded uEPSP is inversely proportional to the spine neck length [5, 8, 9]. Numerical simulations show that the EPSP amplitude/neck length correlation can be explained by variations in synaptic conductance, electrical attenuation through the neck, or a combination of the two [5]. Nevertheless, these models rely exclusively on the passive

electrical attenuation of synaptic inputs through the spine neck assume very high (> 2 GOhm) neck resistance [5], which is at odds with recent spine neck resistance estimations [10, 11]. Furthermore, these simulations suggest that if the neck resistance is low, changes in synaptic conductance mediated by an increase in the number of AMPA receptors could contribute significantly to t-LTP-dependent changes in synaptic strength [5]. Furthermore, AMPA receptor content is one of the major mechanisms underlying LTP (for review see [12]). Hence, we studied the contribution of AMPA receptors to this phenomenon, and our results showed that GluR1 receptor incorporation into the PSD is required for t-LTP induction in spines. What is the role of spine neck shrinkage on the incorporation of AMPA receptors into the PSD and ultimately on t-LTP induction in spines?

Experimental and theoretical studies have indicated that lateral diffusion of AMPA receptors into and out of the spine head can be restricted by the spine neck geometry [13, 14, 15, 16]. In particular, lateral diffusion of AMPA receptors into and out of mushroom spines (long-necked spines) has been shown to be significantly slower than that observed in stubby spines (small-necked spines) [13], which is supported by studies showing reduced diffusion of membrane proteins located in spine necks [17]. In addition, quantitative models using realistic spine morphologies indicate that decreasing the radius and increasing the spine neck length increases the retention of AMPA receptors at the synapse [15], even when their interaction with scaffolding cytoskeletal proteins is neglected [16]. Actin is highly enriched in the spine neck and head [18], and plays an important role in anchoring AMPA receptors in the spine [19] and AMPA receptor trafficking [20], being instrumental for synaptic transmission and plasticity [21, 22, 23]. Hence, to address the role that t-LTP-induced neck shrinkage has on AMPA receptor lateral trafficking to the PSD, and the generation of t-LTP in the activated spines we study actin dynamics. Our results showed that actin polymerization is required for the t-LTP-dependent shrinkage of the activated spine necks and increase in uEPSP amplitude, suggesting that

the induction of t-LTP in spines involves a neck-shrinkage-dependent facilitated diffusion of GluR1 subunits to the spine head where they are incorporated to the PSD.

Micro clusters: a structural and functional modality of synaptic connectivity and plasticity

We found the remarkable result that a *micro cluster* of just two spines during a STDP protocol alter the calcium dynamics and the induction of t-LTP and t-LTD. In fact, the relevance of synaptic *micro clusters* on the input/output properties of pyramidal neurons is also supported by three dimensional electron microscopy and neuronal reconstruction studies that have shown the presence of postsynaptic innervation of the same axon spaced at less than 10 µm in the basal dendrites of L2/3 pyramidal neurons from the medial entorhinal cortex [24], L5 pyramidal neurons from somatosensory cortex [25] and in the distal apical tuft dendrites in stratum lacunosum-moleculare of hippocampal CA1 pyramidal neurons [26]. In addition to having spines innervated by the same axon, it is likely that functional synaptic *micro clusters* can be gated by the convergence of different axons, which could increase the computational power of cortical circuits through a multi-neuronal control of synaptic cooperativity and ultimately the implemented STDP learning rule. Furthermore, it has been shown that orientation selectivity in visual cortex is correlated with the degree of spatial synaptic clustering of co-tuned synaptic inputs within the dendritic field [27], and that functional clusters of dendritic spines separated by less than 10 µm share similar spatial receptive field properties, spontaneous and sensory-driven activity [28]. Interestingly, is has been recently shown in the mouse visual cortex that a single axon can contact clustered synapses in the postsynaptic neuron, but that local clustering is not favoured over widespread spacing of synaptic inputs [29]. In addition, recently it has been demonstrated in L2/3 pyramidal neurons from mouse primary visual cortex that synaptic *micro clusters* reflects the interaction of functionally similar inputs - with similar orientation preference - from different sources – callosal and non-callosal inputs [30], providing functional and anatomical data for the

presence of multi-neuronal control of synaptic cooperativity in a short dendritic segment. Moreover, it has been shown that coactive neighboring synapses drive the maturation of cluster synapses in CA1 pyramidal neurons [31], suggesting that *micro clusters* also play an important role in the development and shaping of network connectivity. Hence, our data and these findings indicate that the presence of synaptic *micro clusters* in the dendrites of pyramidal neurons affect the STDP learning rule, likely providing an efficient strategy for guiding learning and memory, and cognition. Furthermore, the functional consequences of synaptic *micro clusters* in the dendrites of pyramidal neurons and their role in plasticity rules could inspire new strategies for the representation of learning and data efficiency in supervised and unsupervised deep learning algorithms.

References

1. Tanaka JI, Horiike Y, Matsuzaki M, Miyazaki T, Ellis-Davies GCR, Kasai H. Protein synthesis and neurotrophin-dependent structural plasticity of single dendritic spines. *Science* **319**, 1683-1687 (2008).

2. Matsuzaki M, Honkura N, Ellis-Davies GCR, Kasai H. Structural basis of long-term potentiation in single dendritic spines. *Nature* **429**, 761-766 (2004).

3. Oh WC, Hill TC, Zito K. Synapse-specific and size-dependent mechanisms of spine structural plasticity accompanying synaptic weakening. *Proc Natl Acad Sci U S A* **110**, E305-312 (2013).

4. Harvey CD, Svoboda K. Locally dynamic synaptic learning rules in pyramidal neuron dendrites. *Nature* **450**, 1195-1200 (2007).

5. Araya R, Vogels TP, Yuste R. Activity-dependent dendritic spine neck changes are correlated with synaptic strength. *Proc Natl Acad Sci U S A*, (2014).

6. Tonnesen J, Katona G, Rozsa B, Nagerl UV. Spine neck plasticity regulates compartmentalization of synapses. *Nat Neurosci* **17**, 678-685 (2014).

7. Nishiyama J, Yasuda R. Biochemical Computation for Spine Structural Plasticity. *Neuron* **87**, 63-75 (2015).

8. Araya R, Jiang J, Eisenthal KB, Yuste R. The spine neck filters membrane potentials. *Proc Natl Acad Sci U S A* **103**, 17961-17966 (2006).

9. Richardson RJ, Blundon JA, Bayazitov IT, Zakharenko SS. Connectivity patterns revealed by mapping of active inputs on dendrites of thalamorecipient neurons in the auditory cortex. *J Neurosci* **29**, 6406-6417 (2009).

10. Harnett MT, Makara JK, Spruston N, Kath WL, Magee JC. Synaptic amplification by dendritic spines enhances input cooperativity. *Nature* **491**, 599-602 (2012).

11. Jayant K, *et al.* Targeted intracellular voltage recordings from dendritic spines using quantum-dot-coated nanopipettes. *Nat Nanotechnol* **12**, 335-342 (2017).

12. Diering GH, Huganir RL. The AMPA Receptor Code of Synaptic Plasticity. *Neuron* **100**, 314-329 (2018).

13. Ashby MC, Maier SR, Nishimune A, Henley JM. Lateral diffusion drives constitutive exchange of AMPA receptors at dendritic spines and is regulated by spine morphology. *J Neurosci* **26**, 7046-7055 (2006).

14. Holcman D, Triller A. Modeling synaptic dynamics driven by receptor lateral diffusion. *Biophys J* **91**, 2405-2415 (2006).

15. Kusters R, Kapitein LC, Hoogenraad CC, Storm C. Shape-induced asymmetric diffusion in dendritic spines allows efficient synaptic AMPA receptor trapping. *Biophys J* **105**, 2743-2750 (2013).

16. Holcman D, Schuss Z. Diffusion laws in dendritic spines. *J Math Neurosci* **1**, 10 (2011).

17. Shim SH, *et al.* Super-resolution fluorescence imaging of organelles in live cells with photoswitchable membrane probes. *Proc Natl Acad Sci U S A* **109**, 13978-13983 (2012).

18. Frost NA, Shroff H, Kong HH, Betzig E, Blanpied TA. Single-Molecule Discrimination of Discrete Perisynaptic and Distributed Sites of Actin Filament Assembly within Dendritic Spines. *Neuron* **67**, 86-99 (2010).

19. Allison DW, Gelfand VI, Spector I, Craig AM. Role of actin in anchoring postsynaptic receptors in cultured hippocampal neurons: Differential attachment of NMDA versus AMPA receptors. *Journal of Neuroscience* **18**, 2423-2436 (1998).

20. Hanley JG. Actin-dependent mechanisms in AMPA receptor trafficking. *Frontiers in Cellular Neuroscience* **8**, (2014).

21. Kim CH, Lisman JE. A role of actin filament in synaptic transmission and long-term potentiation. *Journal of Neuroscience* **19**, 4314-4324 (1999).

22. Honkura N, Matsuzaki M, Noguchi J, Ellis-Davies GCR, Kasai H. The subspine organization of actin fibers regulates the structure and plasticity of dendritic spines. *Neuron* **57**, 719-729 (2008).

23. Ramachandran B, Frey JU. Interfering with the actin network and its effect on long-term potentiation and synaptic tagging in hippocampal CA1 neurons in slices in vitro. *J Neurosci* **29**, 12167-12173 (2009).

24. Schmidt H, Gour A, Straehle J, Boergens KM, Brecht M, Helmstaedter M. Axonal synapse sorting in medial entorhinal cortex. *Nature* **549**, 469-475 (2017).

25. Kasthuri N*, et al.* Saturated Reconstruction of a Volume of Neocortex. *Cell* **162**, 648-661 (2015).

26. Bloss EB, Cembrowski MS, Karsh B, Colonell J, Fetter RD, Spruston N. Single excitatory axons form clustered synapses onto CA1 pyramidal cell dendrites. *Nat Neurosci* **21**, 353-363 (2018).

27. Wilson DE, Whitney DE, Scholl B, Fitzpatrick D. Orientation selectivity and the functional clustering of synaptic inputs in primary visual cortex. *Nat Neurosci* **19**, 1003-1009 (2016).

28. Scholl B, Wilson, D.E., Fitzpatrick, D. Local Order within Global Disorder: Synaptic Architecture of Visual Space. In: *Neuron*) (2017).

29. Lee WC*, et al.* Anatomy and function of an excitatory network in the visual cortex. *Nature* **532**, 370-374 (2016).

30. Lee KS, Vandemark K, Mezey D, Shultz N, Fitzpatrick D. Functional Synaptic Architecture of Callosal Inputs in Mouse Primary Visual Cortex. *Neuron*, (2019).

31. Lee KF, Soares C, Thivierge JP, Beique JC. Correlated Synaptic Inputs Drive Dendritic Calcium Amplification and Cooperative Plasticity during Clustered Synapse Development. *Neuron* **89**, 784-799 (2016).
