# Supplementary material for: A spike-timing-dependent plasticity rule for dendritic spines
Source: Nat Commun. 2020 Aug 26;11:4276. doi: 10.1038/s41467-020-17861-7 (PMC7449969; doi:10.1038/s41467-020-17861-7)
Supplement: Supplementary file 3 — Reporting Summary [file 41467_2020_17861_MOESM3_ESM.pdf]

nature research

Corresponding author(s): Roberto Araya
Last updated by author(s): Jul 16, 2020

Reporting Summary

Nature Research wishes to improve the reproducibility of the work that we publish. This form provides structure for consistency and transparency in reporting. For further information on Nature Research policies, see Authors & Referees and the Editorial Policy Checklist .

Statistics

For all statistical analyses, confirm that the following items are present in the figure legend, table legend, main text, or Methods section.

n/a	Confirmed	
☐	☒	The exact sample size (*n*) for each experimental group/condition, given as a discrete number and unit of measurement
☒	☐	A statement on whether measurements were taken from distinct samples or whether the same sample was measured repeatedly
☐	☒	The statistical test(s) used AND whether they are one- or two-sided<br>*Only common tests should be described solely by name; describe more complex techniques in the Methods section.*
☒	☐	A description of all covariates tested
☐	☒	A description of any assumptions or corrections, such as tests of normality and adjustment for multiple comparisons
☐	☒	A full description of the statistical parameters including central tendency (e.g. means) or other basic estimates (e.g. regression coefficient) AND variation (e.g. standard deviation) or associated estimates of uncertainty (e.g. confidence intervals)
☐	☒	For null hypothesis testing, the test statistic (e.g. *F*, *t*, *r*) with confidence intervals, effect sizes, degrees of freedom and *P* value noted<br>*Give P values as exact values whenever suitable.*
☒	☐	For Bayesian analysis, information on the choice of priors and Markov chain Monte Carlo settings
☒	☐	For hierarchical and complex designs, identification of the appropriate level for tests and full reporting of outcomes
☒	☐	Estimates of effect sizes (e.g. Cohen's *d*, Pearson's *r*), indicating how they were calculated

Our web collection on statistics for biologists contains articles on many of the points above.

Software and code

Policy information about availability of computer code

Data collection	Electrophysiological data were collected with MultiClamp 700B amplifiers (Molecular Devices).Two-photon images were collected with a custom-built two-photon laser scanning microscope, consisting of 1) a Prairie scan head (Bruker) mounted on an Olympus BX51WI microscope with a 60X, 0.9 N.A. water immersion objective; 2) a tunable Ti-Sapphire laser (Chameleon Ultra-II, Coherent); 3) two photomultiplier tubes (PMT) for fluorescence detection. Fluorescence images were detected with Prairie View 5.4 software (Bruker).
Data analysis	Electrophysiological data were analyzed with Igor Pro 7 (Wavemetrics) software and MATLAB R2019b (MathWorks). The analysis of spine morphology was performed from z-projections of the whole spine using ImageJ 1.52p (neck length) and MATLAB R2019b (MathWorks) (head volume).

For manuscripts utilizing custom algorithms or software that are central to the research but not yet described in published literature, software must be made available to editors/reviewers. We strongly encourage code deposition in a community repository (e.g. GitHub). See the Nature Research guidelines for submitting code & software for further information.

Data

Policy information about availability of data

All manuscripts must include a data availability statement. This statement should provide the following information, where applicable:

- Accession codes, unique identifiers, or web links for publicly available datasets
- A list of figures that have associated raw data
- A description of any restrictions on data availability

The data that support the findings of this study are available in figshare with the identifier doi: 10.6084/m9.figshare.12627422.

Field-specific reporting

Please select the one below that is the best fit for your research. If you are not sure, read the appropriate sections before making your selection.

☒ Life sciences ☐ Behavioural & social sciences ☐ Ecological, evolutionary & environmental sciences

For a reference copy of the document with all sections, see nature.com/documents/nr-reporting-summary-flat.pdf

Life sciences study design

All studies must disclose on these points even when the disclosure is negative.

Sample size	Sample sizes were similar to those generally employed in the field (Araya et al., 2014; Oh et al., 2013; Weber et al., 2016).
Data exclusions	Only neurons for which the injected current to hold the cell at – 65 mV was < 100 pA were included in this study. For the generation of bAP, only action potentials (AP) with amplitude of > 45 mV from threshold to the peak amplitude were considered for analysis. If these conditions were not met, experiments were discontinued immediately. This was applicable to all experimental groups. Thus, no completed experiments were excluded in this study.
Replication	The number of animals used in this study and the number of neurons recorded are clearly stated throughout the Results section. No technical replicates were used in this study.
Randomization	Our animals were not separated into groups.
Blinding	Blinding was not necessary because our animals were not separated into groups.

Reporting for specific materials, systems and methods

We require information from authors about some types of materials, experimental systems and methods used in many studies. Here, indicate whether each material, system or method listed is relevant to your study. If you are not sure if a list item applies to your research, read the appropriate section before selecting a response.

Materials & experimental systems

n/a	Involved in the study	
☒	☐	Antibodies
☒	☐	Eukaryotic cell lines
☒	☐	Palaeontology
☐	☒	Animals and other organisms
☒	☐	Human research participants
☒	☐	Clinical data

Methods

n/a	Involved in the study	
☒	☐	ChIP-seq
☒	☐	Flow cytometry
☒	☐	MRI-based neuroimaging

Animals and other organisms

Policy information about studies involving animals; ARRIVE guidelines recommended for reporting animal research

Laboratory animals	Mice, C57B/6, male and female, postnatal day 14-21. Mice were kept on a 12-hour light/dark cycle at 20-24°C and 40-70% humidity.
Wild animals	This study did not involve wild animals.
Field-collected samples	This study did not involve field-collected samples.
Ethics oversight	These studies were performed in compliance with experimental protocols (13-185, 15-002, 16-011 and 17-012) approved by the Comité de déontologie de l'expérimentation sur les animaux (CDEA) of the University of Montreal.

Note that full information on the approval of the study protocol must also be provided in the manuscript.
